# ACTH-Independent Cushing’s Syndrome Caused by an Ectopic Adrenocortical Adenoma in the Renal Hilum

**DOI:** 10.3390/diagnostics12081937

**Published:** 2022-08-11

**Authors:** Zhixin Hao, Jie Ding, Li Huo, Yaping Luo

**Affiliations:** 1 Department of Nuclear Medicine, Chinese Academy of Medical Sciences and Peking Union Medical College Hospital, Beijing 100730, China; 2 Beijing Key Laboratory of Molecular Targeted Diagnosis and Therapy in Nuclear Medicine, Beijing 100730, China

**Keywords:** Cushing’s syndrome, ectopic adrenocortical adenoma, somatostatin receptor imaging, ^18^F-FDG PET/CT

## Abstract

We report a rare case of Cushing’s syndrome induced by an ectopic adrenocortical adenoma. A 57-year-old woman was diagnosed with adrenocorticotropic hormone (ACTH)-independent Cushing’s syndrome based on clinical manifestation and laboratory information. She was found to have a mass in the left renal hilum via contrast-enhanced computed tomography (CT). The mass was negative, as seen in somatostatin receptor imaging with ^99m^Tc-hydrazinonicotinyl-Tyr3-octreotide (HYNIC-TOC), and showed mild fluorodeoxyglucose (FDG) activity via positron emission tomography (PET)/CT. The results of adrenal venous sampling suggested a left-side adrenal origin of hypercortisolism, possibly secreted by the mass in the renal hilum. Histopathology after surgical resection of the mass confirmed an ectopic adrenocortical adenoma, which was responsible for the patient’s Cushing’s syndrome. During the 8-month follow-up after surgery, no recurrence of Cushing’s syndrome was found.

A 57-year-old woman presenting with weight gain, hypertension, and diabetes for 7 years was admitted to the Peking Union Medical College Hospital. Her vital signs at admission were as follows: blood pressure 176/106 mmHg, pulse 102 beats per minute, weight 84 kg, height 151 cm, and body mass index 36.84 kg/m^2^. Physical examination revealed centripetal obesity, moon face, buffalo hump, and supraclavicular fat pads. She denied any exogenous corticosteroid use.

Laboratory investigations are summarized in Table 1. Plasma cortisol concentration was 27.7 µg/dL in the morning (8 a.m., reference range: 4.0–22.3 µg/dL). The 24 h urinary free cortisol (UFC) was 347.5 µg (reference range: 12.3–103.5 µg). The morning adrenocorticotropic hormone (ACTH) concentration was <5 pg/dL (8 a.m., reference range: 0–46.0 pg/dL). During the 48 h low-dose dexamethasone depression test (LDDST), the morning plasma cortisol was 26.4 µg/dL, and the 24 h UFC was 203.2 µg. Both morning plasma cortisol and 24 h UFC in the LDDST were not suppressed. These findings were consistent with ACTH-independent Cushing’s syndrome.

Abdominal contrast-enhanced computed tomography (CT) demonstrated atrophy of bilateral adrenal glands without any nodules or hyperplasia (Figure 1A, arrowheads). Instead, a mass with mild enhancement measuring 3.1 × 2.7 cm^2^ in the left renal hilum was detected (Figure 1A,B, arrows). Thereafter, she was referred for somatostatin receptor imaging with ^99m^Tc-hydrazinonicotinyl-Tyr3-octreotide (HYNIC-TOC) and ^18^F-fluorodeoxyglucose (FDG) positron emission tomography (PET)/CT to further differentiate the neuroendocrine tumor and its malignancy. ^99m^Tc-HYNIC-TOC single-photon emission computed tomography (SPECT)/CT showed the mass was negative with somatostatin receptor imaging (Figure 1C,D, arrows). In ^18^F-FDG PET/CT (Figure 1E,F, arrows), the mass showed mild radioactivity with the maximum standardized uptake value (SUV_max_) of 2.2, indicating a benign tumor.

As there were no signs of adrenal disease in the current case, ectopic cortisol overproduction by the mass in the renal hilum was suspected; thus, adrenal and renal venous sampling with a cortical hormone assay was carried out (Table 2). The cortisol concentration from the left-side adrenal vein was higher than that of the other veins, suggesting a left-side adrenal origin of hypercortisolism, possibly secreted by the mass in the renal hilum. Then, laparoscopic resection of the mass was performed. The mass was yellowish-brown, and histopathology examination confirmed an ectopic adrenocortical adenoma. On the third postoperative day, the morning plasma cortisol level declined to 9.6 μg/dL, and the plasma ACTH level increased to 8.3 pg/dL. Oral prednisone (20 mg morning/10 mg mid-day/10 mg evening) was given after the operation for 2 weeks, and gradually tapered for an 8-month duration according to clinical symptoms and morning plasma cortisol. No recurrence of Cushing’s syndrome was found at the 4-month and 8-month follow-up postoperatively.

Cushing’s syndrome is a condition of chronic pathological hypercortisolism, which can be either endogenous or exogenous [1]. Endogenous Cushing’s syndrome can be divided into ACTH-dependent, when pathologic ACTH secretion drives glucocorticoid production, or ACTH-independent, when the adrenal glands autonomously secrete excessive glucocorticoid [2]. ACTH-independent Cushing’s syndrome, accounting for approximately 20% of endogenous causes, is more often caused by adrenal adenomas, adrenocortical carcinomas, and rarely, macronodular adrenal hyperplasia or primary pigmented nodular adrenal disease [3]. In our patient, typical manifestations of Cushing’s syndrome, increased cortisol levels, suppressed ACTH levels, and failure of suppression by the LDDST confirmed the diagnosis of ACTH-independent Cushing’s syndrome. However, atrophied bilateral adrenal glands with a mass in the renal hilum was present, which was uncommonly seen in patients with ACTH-independent Cushing’s syndrome. Thus, an ectopic adrenocortical adenoma was suspected.

Ectopic adrenal tissue occurs when there are adrenal rests along the path from gonads to adrenal glands during embryogenesis. Ectopic adrenal tissue is reported in 50% of neonates and 1% of adults [4]. The hormone-secreting status decides the clinical features of ectopic adrenal tissue. Most ectopic adrenal tissue atrophies; occasionally, it may undergo hyperplasia, resulting in the overproduction of cortisol [5]. Ectopic adrenocortical adenoma is an extremely rare cause of ACTH-independent Cushing’s syndrome. The common anatomic sites of ectopic adrenal tissue have been published, including the celiac plexus, kidney, broad ligament, epididymis, and testis [6]. To date, there have been seven cases of ectopic adrenocortical adenoma in the renal hilum reported in the literature [5,7,8,9,10,11,12]. The patients (three males, four females) ranged in age from 27 to 63 years, with the largest diameter measuring 2.3 to 5.3 cm. Of the seven cases, five reported endocrine abnormalities, including Cushing’s syndrome, hyperaldosteronism, and elevation of testosterone, and the remaining two cases were nonfunctioning ectopic adrenocortical adenoma.

The anatomical imaging modalities, such as CT and magnetic resonance imaging (MRI), are important for detecting the ectopic adrenal tumors. However, it is difficult to differentiate an ectopic adrenocortical adenoma from urothelial or renal neoplasms preoperatively, especially in patients lacking hormonal abnormalities [13]. Misdiagnosis of this benign condition in perirenal space may lead to unnecessary radical nephrectomy for the patient. In our patient, ^18^F-FDG PET/CT, a functional imaging modality based on glucose metabolism, was not used in differentiating whether the tumor is responsible for Cushing’s syndrome, but it retained an essential role in determining its malignancy.

The treatment of functioning ectopic adrenal tissue is radical resection, and nonfunctioning ones can be managed with the watch-and-wait approach [14]. However, it is a challenge to confirm the hormone-secreting status of the tumor before surgery. Selective venous sampling is a highly sensitive modality in the localization of various neuroendocrine tumors [15]. Multiple case reports have suggested that adrenal venous sampling may be used to localize excess cortisol between adrenal nodules in patients with ACTH-independent Cushing’s syndrome [16]. The use of venous sampling in localizing ectopic ACTH-independent Cushing’s syndrome is scarcely reported. In our case, we performed combined adrenal and renal venous sampling with a glucocorticoid hormone assay in localizing the ectopic cortisol-producing tumor in the renal hilum.

Functional imaging could be helpful to obviate the invasive venous sampling for the localization of the responsible tumor in ACTH-independent Cushing’s syndrome. Adrenal scintigraphy with radiolabeled cholesterol analogs, such as ^131^I-6-β-iodomethyl-norcholesterol and ^75^Se-selenomethyl-norcholesterol, might be helpful to diagnose the ectopic functional adrenocortical tumor, although it is not widely used [17,18]. CXCR4, a G-protein-coupled chemokine receptor expressed on the surface of the cell membrane, was reported to be upregulated in adrenal adenoma [19]. Recently, PET/CT with ^68^Ga-pentixafor, a CXCR4-specific PET tracer, was reported to exhibit great potential for identifying functional adrenocortical adenomas [20,21]. We think it might also be effective for the localization and identification of the ectopic functional adrenocortical tumor, such as in the current case. Ectopic ACTH syndrome, usually caused by small-cell lung carcinoma, pulmonary or thymic neuroendocrine neoplasms, pancreatic neuroendocrine tumors, etc., is a rare cause of ACTH-dependent Cushing’s syndrome [22]. However, somatostatin receptor imaging, helpful in detecting ectopic ACTH syndrome, has a limited role regarding the identification of the responsible tumor in ACTH-independent Cushing’s syndrome [23].

In summary, this case report provides a rare case of ectopic adrenocortical adenoma in the renal hilum for clinicians. Selective venous sampling, adrenocortical scintigraphy, and potentially, ^68^Ga-pentixafor PET/CT, may be helpful in diagnosis and confirming the origin of the tumor.

## Figures and Tables

**Figure 1:**
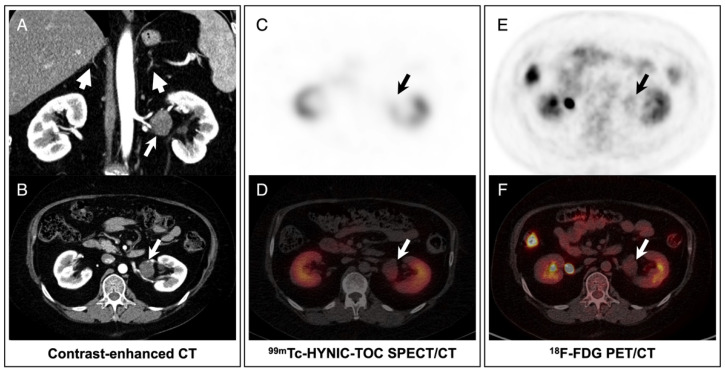
Imaging analysis of the patient. (**A**,**B**) Abdominal contrast-enhanced CT showed bilateral adrenal glands were atrophic without any nodules or hyperplasia (arrowheads), and a mass with mild enhancement in the left renal hilum (arrows). (**C**,**D**) ^99m^Tc-HYNIC-TOC SPECT/CT showed the mass was negative with somatostatin receptor imaging (arrows). (**E**,**F**) ^18^F-FDG PET/CT revealed mild FDG activity of the mass (arrows).

**Table 1:** Laboratory results of the patient.

Variables	Reference Range	At Admission	After Surgery	4-Month Follow-Up	8-Month Follow-Up
24 h UFC (µg)	12.3–103.5	347.5	93.4	NA	NA
8 a.m. cortisol (µg/dL)	4.0–22.3	27.7	9.6	4.1	2.2
8 a.m. ACTH (pg/dL)	0–46.0	<5	8.3	9.5	8.0
LDDST 24 h UFC (µg)	12.3–103.5	203.2	NA	NA	NA
LDDST cortisol (µg/dL)	4.0–22.3	26.4	NA	NA	NA

UFC: urinary free cortisol; ACTH: adrenocorticotropic hormone; LDDST: low-dose dexamethasone suppression test; NA: not available.

**Table 2:** Laboratory results of venous sampling.

	Left Adrenal Vein	Left Renal Vein (Proximal)	Left Renal Vein (Distal)	Right Renal Vein (Distal)	Peripheral Vein
Cortisol (µg/dL)	28.1	21.1	18.7	19.3	23.8

## Data Availability

Not applicable.
